# Lesions in canine stifle joints due to trochleoplasties as treatment for medial patellar luxation

**DOI:** 10.4102/jsava.v86i1.1245

**Published:** 2015-07-01

**Authors:** Johannes Hans van der Zee

**Affiliations:** 1 Bridge Animal Hospital (Pty) Ltd, Pretoria, South Africa

## Abstract

Lesions in canine stifle joints after previous trochleoplasty surgery were documented. In four clinical cases arthrotomies were performed due to stifle pain after previous trochlear deepening procedures. A small area of hyaline cartilage remained in the groove of the stifles in cases where previous wedge trochleoplasties had been performed. All of the stifles had significant areas of eburnation on the axial aspect of the medial trochlear ridge. The stifle joints of a dog that was euthanased due to severe irreversible osteo-arthritis were photographed. The dog had undergone previous surgery for patellar luxation and cranial cruciate ligament ruptures. The trochlear grooves in this dog had almost no visible articular cartilage left.

## Introduction

Medial patellar luxation (MPL) is one of the most common orthopaedic conditions in small animal surgery (Alam *et al.* 2007; Bound *et al.* 2009). Although the anatomical deformities associated with MPL are well described, the exact pathogenesis is still not clear (Hulse 1993; Kowaleski, Boudrieau & Pozzi 2012). Despite the fact that there is no objective standard for the depth of the trochlear sulcus, one of the most frequent surgical treatments recommended in the literature is deepening of the trochlear sulcus (Kowaleski *et al.* 2012; Talcott, Goring & De Hann 2000). Variations of the wedge or block trochleoplasty are the most frequently recommended techniques (Bound *et al.* 2009; Johnson *et al.* 2001; Kowaleski *et al.* 2012; Schulz 2007; Talcott *et al.* 2000).

During a review of the literature on MPL in animals the author could find no evidence in 56 references documenting any criteria to judge or quantify the adequacy of trochlear depth, nor any evidence that the patella luxates due to a shallow trochlear sulcus (Van der Zee 2010). A cartilage-sparing technique has been described for young dogs (Flo 1969). Alternative methods to a trochleoplasty have been described (Linney, Hammer & Shott 2011). In the human medical literature trochleoplasties are currently performed only in patients with severe dysplasia, where stability of the patellofemoral joint cannot otherwise be obtained (Iliadis, Jaiswal & Khan 2012).

In this case report the author has documented lesions on the trochlear grooves in dogs after previous trochleoplasty surgeries.

## Materials and methods

The appearance of trochlear lesions after trochleoplasty are described. Four clinical cases were referred due to recurrent pain after previous trochleoplasties had been performed. In one case there was recurrence of MPL, but the other three had no obvious cause for the pain on clinical examination.

**FIGURE 1:**
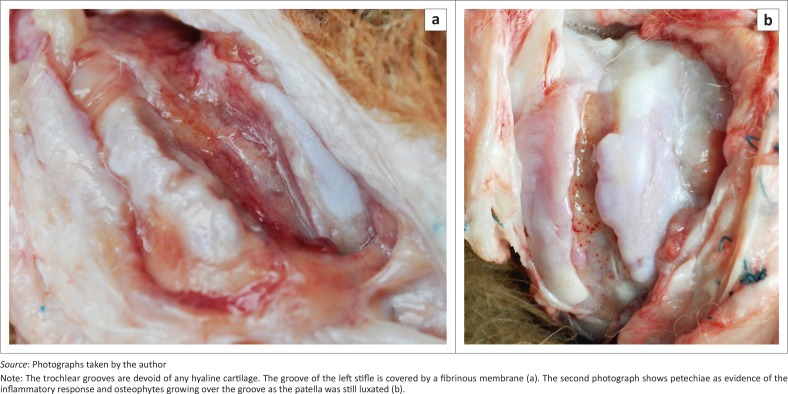
(a, b) Photographs of the stifles of the dog that was euthanased.

Photographs are presented of a 5-year-old chow that had been euthanased due to severe osteo-arthritis after having had surgery for MPL and cranial cruciate ligament injuries. This dog was presented due to severe pain in the stifle joints with resultant inability to stand up on the hind legs. Radiographs demonstrated severe bilateral degenerative changes in both stifle joints. Surgical procedures that had been performed were rasp sulcoplasties and extra-articular stabilisation sutures. It was decided that the only reasonable treatment for this dog would have been total stifle joint replacements, but the owner elected euthanasia (Figure 1).

All of the dogs had a full orthopaedic examination, radiography and anaesthetic risk evaluation before the stifle arthrotomies were performed. Pre-anaesthetic sedation in preparation for surgery was achieved with morphine (Morphine sulphate, Fresenius Kabi) (0.5 mg/kg intravenously [IV]) and Valium (Pax, Pharmacare) (0.3 mg/kg, IV). Pre-operatively meloxicam (Metacam, Boehringer Ingelheim) (0.2 mg/kg subcutaneously) was administered, followed by oral administration of meloxicam (Petcam, Ciplavet) (0.1 mg/kg every 24 hours) for 7 days. Amoxicillin/clavulanic acid (Augmentin, GlaxoSmithKline) (20 mg/kg IV every 8 hours) was administered for a total of 24 hours. Anaesthesia was induced with propofol (Propofol 1%, Fresenius Kabi) (4 mg/kg – 6 mg/kg, IV, titrated to effect) and maintained with sevoflurane (Sojourne, SafelinePharm) (3%) in 100% oxygen. Intravenous balanced electrolyte solution (Sabax Ringer-Lactate, Adcock Ingram) (10 mL/kg/h, IV) was administered during the procedure.

## Results

**FIGURE 2:**
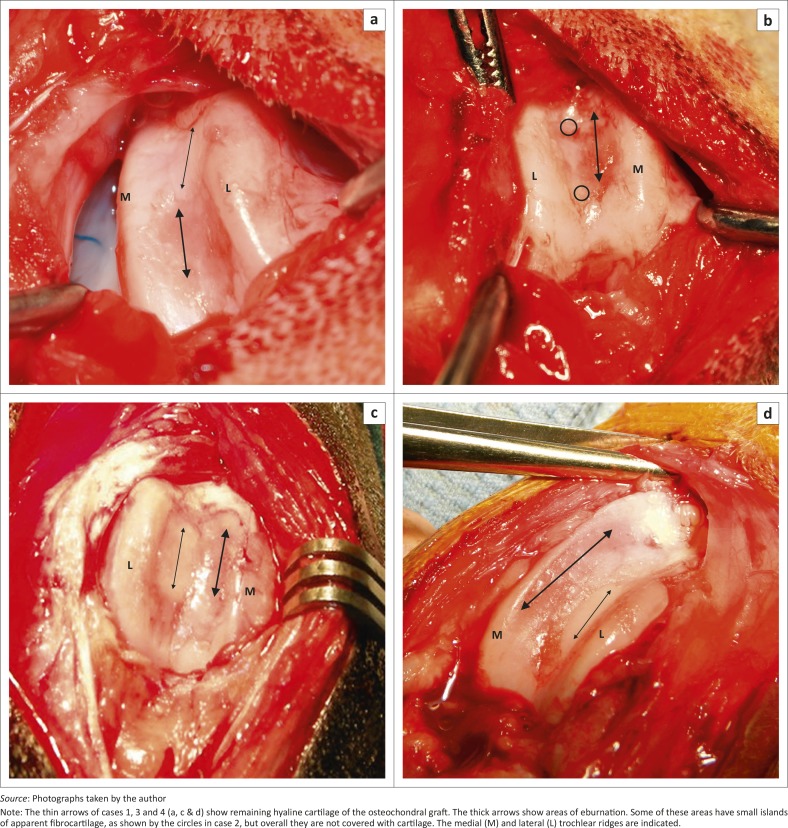
(a-d) Photographs of the four clinical cases.

The history, signalment, clinical examination and radiographic findings as well as intra-operative findings and medium-term outcomes were recorded and are presented in Table 1 and Figure 2.

**TABLE 1:** Signalment, findings during arthrotomy, treatment and follow-up of the four clinical cases.

Number	Breed, age, weight	History	Arthrotomy findings	Treatment	Telephonic follow-up time and result
1	Min doberman cross, 7 years, 5 kg	Previous wedge trochleoplasty 3 years ago. Had improved but got worse again, 6/10 lameness. Patella in place. Mild DJD of the stifle on radiographs	Very little left of the hyaline cartilage. Areas that appear as fibrocartilage. Areas of eburnation on the axial aspect of the medial trochlear ridge and on the patella	Release and re-implantation of the sartorius, lateral capsulorrhaphy	12 months. Marked improvement, but still a 2/10 lameness and occasional need for anti-inflammatories
2	Labrador, 5 years, 25 kg	Previous abrasion arthroplasty 2 years ago, 6/10 lame. No instability. Patella reduced. Moderate DJD on radiographs	Areas that appear as fibrocartilage, large area of eburnation on the axial aspect of the medial trochlear ridge. Corresponding area of eburnation on the patella. Partial CCL tear	Release and re-implantation of the sartorius, lateral capsulorrhaphy. Tibial tuberosity advancement with lateralisation of the crest	16 months. Marked improvement, with only 2/10 lameness
3	Staffordshire bull terrier, 3 years, 18 kg	Wedge trochleoplasty 3 months earlier. Grade 1 reluxation 6 weeks post-op. 8/10 lameness. Mild DJD of the stifle on radiographs	The previous wedge resection had healed. There were however two ridges of exposed bone where the wedge had been recessed. There also was a large area of eburnation on the patella	Release and re-implantation of the sartorius, lateral capsulorrhaphy	6 months. Significant improvement after surgery with hardly any lameness. Died from a snakebite 3 months post-op. and re-examination not possible
4	Boxer, 18 months, 22 kg	Wedge trochleoplasty and tibial crest transpositioning had been done 6 months ago; improved but then deteriorated again to current 7/10 lameness. Moderate DJD of the stifle on radiographs Mild instability of the cranial cruciate ligament. Patella reduced	The previous wedge resection had healed, but a very small area of hyaline cartilage had remained. There was a large area of eburnation on the axial aspect of the medial trochlear ridge, with a corresponding area of eburnation on the patella. Partial cranial cruciate ligament tear	Release and re-implantation of the sartorius, lateral capsulorrhaphy. Tibial tuberosity advancement with lateralisation of the crest	3 months. Marked improvement. Only occasional lameness. 1/10, anti-inflammatories not required

All four clinical cases had areas of eburnated bone on the axial aspect of the medial trochlear ridge, with concurrent cartilage eburnation on the medial aspect of the patella. The exact location of lesions on the trochlea in clinical cases of MPL is not clearly defined in the literature. In the author's experience of more than 250 clinical cases the area of erosion is on the proximal and abaxial aspect of the medial trochlear ridge and not on the axial aspect. This is also the case in a photograph of a case described in an article documenting the erosion on the patella of 145 stifles with MPL (Daems, Janssens & Béosier 2009).

The stifle joints of the dog that had been euthanased showed severe pathology of the trochlear grooves and of both patellae. Although most of the findings noted here could have been diagnosed with arthroscopy, the author did not have access to this modality at the time of the presentation of these cases. According to the histories obtained, these wedge trochleoplasties had been performed in accordance with recommended techniques.

## Discussion

Lesions of the trochlear sulcus after previously performed trochleoplasties are described. The author could not find any previous reports of these lesions.

Any insult to the joint cartilage results in degenerative changes in the cartilage and a resultant inflammatory response (Mankin 1982). Studies have shown the formation of fibrocartilage in subchondral defects (Shapiro, Koide & Glimcher 1993). However, these studies were done in cases with normal opposing cartilage.

Hyaline articular cartilage cannot heal or reform to its original properties (Mankin 1982). Whatever modification is made during the trochleoplasty procedure, it is inevitable that the cartilage surface is cut and that exposed areas of cartilage-devoid bone will be created. Although this exposed bone, if cut to the subchondral level, will result in creation of patches of fibrocartilage, this repair does not have the same qualities as that of hyaline cartilage (O’ Driscoll 1998). Another problem is that the patella and parapatellar cartilages are not contoured to the new shape of the groove, thereby resulting in abnormal pressure areas that will lead to further cartilage degradation (Daems *et al.* 2009). Some authors recommend cutting the patella to fit to the newly formed groove (Vasseur 2003), but this will form another area on the patella without cartilage that will contribute to the joint degeneration and inflammation.

Lesions on the patellae were identified as present in 93 of 145 stifles with MPL in a study by Daems *et al.* (2009). The cartilage of the patella cannot heal as the lesions do not reach the subchondral bone. Only lesions that reach the subchondral bone can heal by formation of new fibrocartilage (O’ Driscoll 1998). If the medial force on the patella is not adequately neutralised, even a small defect caused by the mechanical force will fail to heal and will degenerate further over time (O’ Driscoll 1998).

Damage to hyaline cartilage will always lead to osteo-arthritis. It has been shown that the degenerative joint disease (DJD) in stifles with MPL progressed despite surgery, mainly with trochlear deepening procedures (Roy *et al.* 1992). Techniques such as trochlear sulcoplasty may prevent accurate patellofemoral articulation and normal articulation pressure (Moore & Banks 1989). It is possible that due to the fact that most of the clinical cases are in miniature breeds, the consequences are just not very severe or are just not noted, similar to osteo-arthritis frequently being missed in cats (Guillot *et al.* 2012).

MPL is becoming more prevalent in large-breed dogs (Gibbons *et al.* 2006). The severity of disease was shown to be more severe in large-breed dogs, and surgical recommendations must be made carefully. It is not possible to do a wedge or even a block trochleoplasty without creating areas of exposed bone that, when opposed to denuded patellar bone, will not form new fibrocartilage. Due to the inability of hyaline cartilage to heal or regenerate, the tendency in human articular surgery is to interfere with the hyaline cartilage as little as possible (Dejour, Walch & Nove-Josserand 1994).

Although the DJD and pain in two of the clinical cases described here could be ascribed to the presence of concurrent cranial cruciate ligament disease, the lesions of the patellar groove and patella per se were likely to contribute to the symptoms. Lowering the surface of the trochlea increases the required extension forces by reducing the lever-arm forces required for leg extension, and increases the pressure from the patella on the groove (Campbell *et al.* 2010).

The common recommendation of different trochleoplasty techniques has been questioned (Linney *et al.* 2011). To the author's knowledge, the normal depth and shape of the canine trochlear groove has not yet been described. In cases where the trochlear groove has never formed, it will be necessary to do some kind of plasty, but to the author's knowledge the incidence of dysplastic grooves has not been reported. In the experience of the author with more than 250 cases with patella luxation, true dysplasia is very rare, with the majority of cases having either a normal groove or a shallow groove due to changes of the medial trochlear ridge. What is important, however, is to check that the alignment of the extensor muscles of the stifle with the tibial crest lies over the centre of the trochlear groove.

The main aim of treatment in the four cases reported here was to shift the medially directed pressure coming from the patella away from the eburnated areas on the axial surface of the medial trochlear ridge to areas with cartilage. This was done by releasing the medial tension on the patella by release and proximal reimplantation of the cranial head of the sartorius muscle, medial fascia release and lateral capsule imbrication. The sartorius muscle was cut at its insertion on the patella and freed from its attachment to the quadriceps muscle halfway down its entire length. The distal end was then sutured to the medial fascia of the quadriceps muscle at a point at 20% of its length, proximal to the patella. The author prefers to suture the insertion of the sartorius muscle back to the quadriceps muscle, as it is necessary for adduction of the limb.

Although this technique has not been documented yet, it is a modification of a similar procedure described by Horne in 1979. The author has treated more than 130 consecutive cases with this technique. In cases numbers 2 and 4 in this study a tibial tuberosity advancement and lateralisation was also performed due to concurrent cranial cruciate ligament injury. The lameness did not resolve in all cases, but it improved significantly. The chronic pain was probably due to the persistent DJD from the remaining lesions in the joints.

## Conclusion

Deepening procedures of the trochlear groove can lead to new areas of cartilage erosions that will perpetuate DJD. In the opinion of the author the indication for deepening procedures of the trochlear groove needs to be more specific, with objective, measurable criteria. The normal depth of the trochlear sulcus for the affected breeds needs to be determined in order to provide objective recommendations.
